# Pathophysiology of COVID-19: Everywhere You Look You Will See ACE_2_!

**DOI:** 10.3389/fmed.2021.694029

**Published:** 2021-08-27

**Authors:** Vladimir L. Cousin, Raphael Giraud, Karim Bendjelid

**Affiliations:** ^1^Intensive Care Division, Geneva University Hospitals, Geneva, Switzerland; ^2^Geneva Hemodynamic Research Group, Geneva, Switzerland; ^3^Faculty of Medicine, Geneva University Hospitals, Geneva, Switzerland

**Keywords:** pathophysiology, COVID-19 pandemic, angiotensin-converting enzyme, SARS – CoV – 2, bradykinin - analogs and derivatives, lung injury

## Abstract

Angiotensin converting enzyme 2 (ACE_2_) seems to be a central actor in the pathophysiology of SARS-Cov-2 infection. First, it acts as the receptor for the virus and permits its attachment to cells expressing ACE_2_. Second, the relative deficiency of ACE_2_ during infection could be linked to several clinical features encountered during the disease, like ARDS and coagulation abnormalities. This study explores the strong link between ACE_2_ and the majority of risk factors for the severe evolution of COVID-19. It seems that all these risks factors are linked to an increased level of ACE_2_ and/or imbalance in ACE/ACE_2_.

## Introduction

COVID-19 is a worldwide progressing pandemic caused by the Severe Acute Respiratory Syndrome Coronavirus type 2 (SARS-CoV-2). Present pneumonia (pneumonitis) is an acute respiratory illness associated with a new droplet-borne SARS-CoV-2, which caused the global population to be put under lockdown, with many patients clustered in hospitals. It has a wide spectrum of clinical severity, ranging from asymptomatic to fatal outcomes. The virus possesses 4 main structural proteins: spike, membrane, envelope, and nucleocapsid (1). Of special interest for our discussion is the spike protein, which attaches to human cells through the angiotensin converting enzyme 2 (ACE_2_) (2). Such a mechanism is common in the two SARS virusus (1, 3). Following host cell binding, with the priming by the transmembrane serine protease 2 (TMPRSS2) and other proteins, the virus and cell membrane fuse, enabling the virus to enter the cell and infect it (1). These interactions with the SARS-CoV-2, ACE_2_ play a crucial role in viral pathology since it is the viral receptor that provides the opportunity for the virus to invade cells expressing such enzymes. Other than this role as a viral receptor, the physiological role of ACE_2_ is crucial, as it reduces angiotensin 2 levels (breakdown) and so plays a role as a regulator in the renin angiotensin system balance.

## Ace_2_ Role and Impact of Dysregulation

ACE2 has been known for 20 years and has brought major insight to understanding of the complex renin-angiotensin system (RAS) (4, 5). ACE_2_ is an enzyme, a carboxypeptidase, which cleaves angiotensin 1 into angiotensin 1–9 and angiotensin-2 into angiotensin 1–7 (6). Through those reactions, ACE_2_ plays a crucial role as a regulator of the RAS. If they are both peptidases, ACE and ACE_2_ have a different active site and the two enzymes manage to counterbalance each other (5, 7). ACE_2_ reduces the level of angiotensin 2, thereby reducing its capacity of action as a potent vasopressor and pro-inflammatory signal (6). Furthermore, ACE_2_ products, mostly angiotensin 1–7, act through a specific pathway to counter angiotensin 2 and mitigate the action of the ligation between angiotensin 2 and the receptor AT1R. The two receptors are of main importance for these pathways. The AT2 receptor (AT2R), Mas receptor (MasR) and induced vasodilation have anti-fibrotic and anti-inflammatory properties (8). Another role of ACE_2_ is the cleavage of other bioactive peptides than angiotensin and especially bradykinin, more precisely des-Arg-Bradykinin (9, 10). Bradykinin, especially des-Arg-Bradykinin, binds bradykinin receptor B1 (BKB1R). Ligation to BKB1R induces the release of inflammatory chemokines. It has a role in vasodilatation, cellular proliferation, and fibrosis (9, 11). There is also an intricate role of bradykinin with the coagulation and the complement activation (9, 12). All the present findings emphasize the particular vascular features of COVID-19 disease. In this regard, the authors believe that Acute Vascular Distress Syndrome (acronym “AVDS”) seems to be more appropriate for COVID-19 than the usual ARDS (acute respiratory distress syndrome) acronym (13).

ACE_2_ is widely expressed inside organs, including, in the lungs, cardiovascular system, gut, kidneys, central nervous system, and adipose tissue. As a result of these roles, it is currently thought that ACE_2_ plays a major role as a cardioprotective actor. It has been linked to several situations of heart failure, hypertension, pulmonary hypertension, diabetes, and acute respiratory distress syndrome (5). ACE_2_ has well-described associations with better outcomes in the case of cardiac dysfunction and is linked to cardiac fibrosis and inflammation in several studies (5). Moreover, angiotensin 1–7 seems to play a protective role in diabetic cardiomyopathy and nephropathy (14, 15). In this regard, increased expression of ACE_2_ protects against hypertension (5). ACE_2_ is also strongly involved in acute pulmonary lesions and fibrosis, as a protector, by inducing an imbalance against RAS hyper activation (5, 16).

As we have seen, the cardioprotective effect of ACE_2_ could be attributed to several mechanisms, including the degradation of angiotensin 1 and angiotensin 2, and so limits activation of AT1R, production of angiotensin 1–7 and 1–9, which have a direct cardioprotective role, and also contributing to the degradation of bradykinine and thereby limiting its pro-inflammatory effects.

## Ace_2_ and COVID-19 Infection Severity

There are several described risk factors for the severe evolution of COVID-19, summarized in Table 1. The earliest studies on the subject clearly showed such an association (17, 18). An important fact to underline is the strong link between ACE_2_ and the majority of these risk factors. It seems that all these risks factors are linked to an ACE_2_ increased level and/or imbalance in ACE/ACE_2_. A study dosing the soluble ACE_2_, as a surrogate marker for the level of ACE_2_, showed significantly increased amounts of soluble ACE_2_ in patients with diabetes, heart failure, older age, and male gender (19). It could be suggested that such a situation, with an increased ACE_2_ at baseline due to an already imbalanced RAS, may be prone to more severe SARS-CoV-2 infection (20). Hypertension was very early reported as a risk factor for fatal outcomes in COVID-19 (21, 22). Hypertension may be linked to a state of hyper activation of ACE_2_ to counter regulate the high blood pressure, meaning these patients have a higher number of targets for the virus to attach to. Male gender is also a risk factor for more severe COVID-19 (23). It could be linked to the potential impact of sex hormones on ACE_2_ expression, RAS balance, or a difference in the proportion of comorbidities (24). As an illustration, ACE_2_ could be found in higher concentrations in the sputum of asthmatic men or plasma of male patients with cardiac failure (25, 26). Patients of Black ethnicity are also at risk of severe COVID-19 and death from COVID-19 (27, 28). However, such risk is currently not well understood, as even if a higher proportion of patients are hospitalized or have fatal outcomes, patients of Black ethnicity seem to have a higher risk when adjusted for multiple factors (28). Patients from Black ethnicities often have comorbidities such as diabetes or hypertension, risk factors that have already been described for severe COVID-19 (28). Moreover, social disparities such as disadvantages in housing and more globally systemic structural disadvantages put such a population at higher risk. This may explain the increased risk for patients of Black ethnicities. Another reason for these comorbidity and population characteristics is that a potential risk factor for severe COVID-19 is decreased levels of ACE_2_ at baseline.

**Table 1:** Risk factors for severe SARS-CoV-2 infection.

**Overweight**
**Black ethnicity**
**Diabetes mellitus**
**Hypertension**
**Chronic heart failure**
**Male**

*Table depicting major risks factors for severe COVID-19, all of them linked to a baseline situation at risk linked to increased level ACE2 and/or baseline imbalance in ACE/ACE2 index. Clockwise from the top, these include being overweight, patients of Black ethnicity, diabetes mellitus, hypertension, cardiac failure, and male gender*.

An interesting paper by Peters et al. on COVID-19 related gene expression in the sputum in asthmatic patients discusses these points of view (25). Patients of Black ethnicities seem to have an increased expression of ACE_2_ in sputum cells, along with male gender and diabetic patients. This raises the question of specific risk linked to increased ACE_2_ in Black people. However, the link between COVID-19, ethnicity, and prognosis remains difficult to prove, as underlined by the recent study of Colarusso et al. (9). The authors showed that if Black ethnicities were admitted to ICU they died more frequently during the first “wave”. This was not obvious during the second wave, as the authors only had an increased risk of ICU admission without an increase in mortality (29). These papers underline the already discussed risk factors of male gender and diabetes. Being overweight and obese, are also risk factors. It is common knowledge that obesity is linked to hypertension, diabetes, and heart failure, as already discussed. Moreover, obese patients may have a pro-inflammatory state, predisposing them to a higher impact of RAS imbalance (11).

An important question is the place of treatment for hypertension targeting the ACE, including ACE-inhibitors of Angiotensin receptor blockers. Such treatment is deeply linked to the RAS and has been used in a large proportion of patients with hypertension, diabetes, or obesity, as all these comorbidities are often associated. Interestingly, these medications were linked to less severe disease (and even better outcome) in pneumonia related to influenza infection and so raised the question of their role in COVID-19 infection (30–32). However, such benefits for COVID-19 patients undergoing pneumonia treatment are currently unproven and unfounded (33, 34).

In focussing on ACE_2_, we see that all these risk factors could be linked to the more severe features of COVID-19 disease. There are populations for which specific research needs to be done in order to investigate the impact of ACE_2_ and therapy aiming at restoring the RAS balance.

## Impact of COVID-Linked Ace_2_ Dysregulation

**Figure 1:**
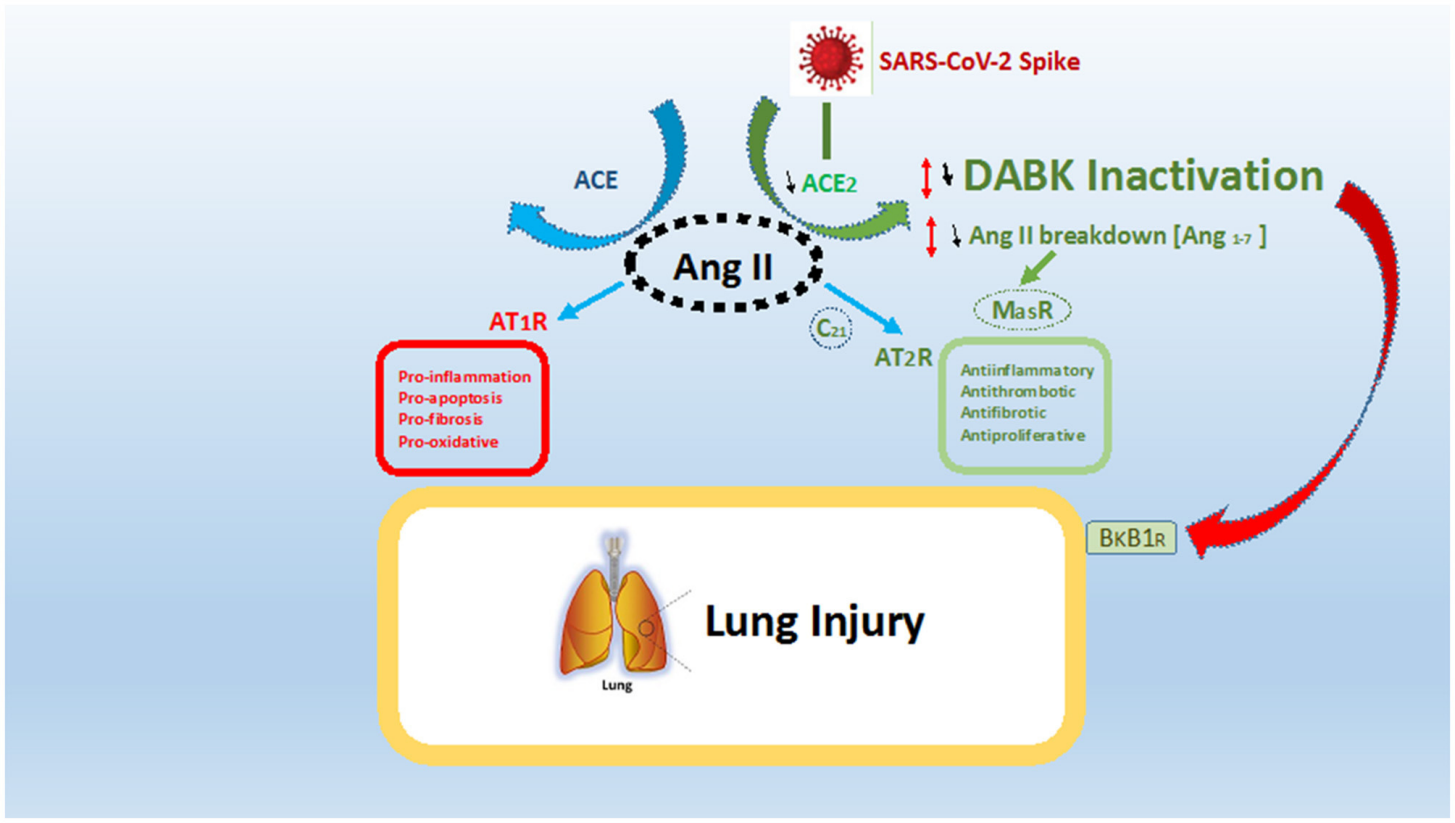
ACE/ACE2 imbalance and effect in case of SARS-CoV-2 infection. SARS-CoV-2 spike protein ligates angiotensin conversion enzyme 2 (ACE_2_) and leads to a relative deficiency in ACE_2_. Such deficiency leads to both an imbalance in the ACE/ACE_2_ system and an over activation of the ACE pathway with over production of angiotensin 2 (Ang II) ligating the receptor AT1R. The present fact leads to a global pro-inflammatory, pro-fibrotic, and pro-oxidative state (in red). ACE_2_, through degradation of Ang II to angiotensin 1–7, leads to both a decrease in Ang II levels and stimulation of anti-inflammatory and anti-fibrotic pathways *via* AT2R and MasR (in green). Moreover, ACE_2_ inactivate des-Arg-Bradykinin (DABK), which in the case of ligation to receptor Bradykinine-B1-receptor (BkB1R) leads to inflammation and vascular permeability (red arrow). In this regard, the final result of ACE_2_ deficiency is both the promotion of lung inflammation and a decrease in lung anti-inflammatory effects, inducing SARS-CoV-2 acute respiratory syndrome.

In case of a SARS Cov-2 infection, there is ligation of the virus to ACE_2_ with its spike protein. Such ligation to ACE_2_ caused its internalization and down-regulation following SARS-CoV-2 cellular entry (6, 11). In such a case, the decrease in ACE_2_ activity creates an imbalance in signaling by ACE_1_ and ACE_2_ due to deficiency in ACE_2_. As we discussed earlier, such decreases in ACE_2_ lead to an imbalance, where angiotensin 2 is the main actor, as shown in Figure 1.

A higher level of angiotensin 2 is linked to the pro-inflammatory and pro-fibrotic situation after ligation to AT1R. Moreover, a severe decrease in ACE_2_ has a double effect: first, there is a decrease of Angiotensin 1-7, lowering activation of the MasR or AT2R, which impedes anti-inflammatory and anti-proliferation pathways (4, 5, 7). Second, there is an increase of D-Arg bradykinin, with inflammatory and vasoactive properties through BKB1R (9, 11). This finally leads the RAS equilibrium to imbalanced conditions characterized by pro-inflammatory, pro-apoptosis, and pro-oxidative states. Moreover, the deregulation of RAS, especially in patients who already are in a state of ACE/ACE_2_ imbalance, could lead to more severe COVD-19 (5).

If we look specifically at the lung, ACE_2_ deficiency is known to be linked to acute lung injury. It has a role in limiting the angiotensin 2 hypoxic vasoconstriction but also pro-fibrotic and inflammatory effect, both meet in case of severe SARS-CoV-2 acute respiratory distress syndrome (1, 7, 11). Diminished levels of ACE_2_ and an imbalance in the ACE/ACE_2_ system could be a major factor in the outcome of COVID-19, as previously noted in laboratory experimentation on acute lung injury. The effect of imbalance could also explain the significant impact of severe SARS-CoV-2 infection on several systems, especially cardiovascular, including systemic endothelitis, renal, and coagulation (1, 7, 11, 35, 36).

In other pulmonary infections, ACE_2_ and angiotensin II were also studied and potentially linked to disease severity. In particular, influenza infection, in which a link between ACE_2_ deficiency and lung injury severity has been observed (37, 38). Moreover, increased levels of Angiotensin-II in a patient with severe influenza infection or coxsackie virus, emphasized the key role of ACE_2_ in other viral lung infections leading to ARDS (39). The role of the RAS system in potential lung cytokine storm and fibrosis could explain such an association between ACE_2_, the RAS system, and viral ARDS (39, 40).

To conclude, ACE_2_ seems to be a central actor in the pathophysiology of SARS-Cov-2 infection. First, it acts as the receptor for the virus and permits its attachment to cells expressing ACE_2_. Second, the relative deficiency of ACE_2_ during infection could be linked to several clinical features encountered during the disease, such as ARDS, vascular inflammation, and coagulation abnormalities (41, 42). Further research is needed to better understand the role of ACE_2_ in virus pathophysiology and ACE_2_ as a potential therapeutic target. In this regard, a soluble form of ACE_2_ may both slow viral entry into cells by competitively binding with SARS-CoV-2 and protect the lung from injury through its unique enzymatic function (2).

## Author Contributions

KB contributed to the conception and design of the review and Figure 1. VC design and first draft. RG designed Table 1. All authors contributed to manuscript revision, and read and approved the submitted version.

## Conflict of Interest

The authors declare that the research was conducted in the absence of any commercial or financial relationships that could be construed as a potential conflict of interest.

## Publisher's Note

All claims expressed in this article are solely those of the authors and do not necessarily represent those of their affiliated organizations, or those of the publisher, the editors and the reviewers. Any product that may be evaluated in this article, or claim that may be made by its manufacturer, is not guaranteed or endorsed by the publisher.
